# Phylogeny of the ciliate family Psilotrichidae (Protista, Ciliophora), a curious and poorly-known taxon, with notes on two algae-bearing psilotrichids from Guam, USA

**DOI:** 10.1186/s12862-019-1450-z

**Published:** 2019-06-18

**Authors:** Xiaotian Luo, Jie A. Huang, Lifang Li, Weibo Song, William A. Bourland

**Affiliations:** 1 Key Laboratory of Aquatic Biodiversity and Conservation of Chinese Academy of Sciences, Institute of Hydrobiology, Chinese Academy of Sciences, Wuhan, 430072 China; 2 Department of Biological Sciences, Boise State University, Boise, ID 83725 USA; 3 Marine College, Shandong University, Weihai, 264209 China; 4 Institute of Evolution & Marine Biodiversity, Ocean University of China, Qingdao, 266003 China

**Keywords:** Algal symbionts, Biodiversity, Biogeography, Ciliate, *Hemiholosticha*, Protist, 18S rRNA gene

## Abstract

**Background:**

The classification of the family Psilotrichidae, a curious group of ciliated protists with unique morphological and ontogenetic features, is ambiguous and poorly understood particularly due to the lack of molecular data. Hence, the systematic relationship between this group and other taxa in the subclass Hypotrichia remains unresolved. In this paper the morphology and phylogenetics of species from two genera of Psilotrichida are studied to shed new light on the phylogeny and species diversity of this group of ciliates.

**Results:**

The 18S rRNA gene sequences of species from two psilotrichid genera were obtained. In the phylogenetic trees, the available psilotrichid sequences are placed in a highly supported clade, justifying the establishment of the family Psilotrichidae. The morphology of two little-known species, packed with green algae, including a new species, *Hemiholosticha kahli* nov. spec., and *Psilotrichides hawaiiensis* Heber et al., 2018, is studied based on live observation, protargol impregnation, and scanning electron microscopy. Both species are easily recognized by their green coloration due to the intracellular algae, and a comprehensive discussion as to the possible roles of the intracellular algae is provided.

**Conclusions:**

The 18S rRNA gene phylogeny supports the morphological argument that *Hemiholosticha*, *Psilotrichides* and *Urospinula* belong to the same family, Psilotrichidae. However, the single-gene analysis, not surprisingly, does not resolve the deeper relationships of Psilotrichidae within the subclass Hypotrichia. Two little-known psilotrichid genera with green algae were collected from the same puddle on the island of Guam, indicating a high species diversity and broader geographic distribution of this group of ciliates than previously supposed. Phylogenetic inferences from transcriptomic and/or genomic data will likely be necessary to better define the systematic position and evolution of the family Psilotrichidae. Further studies are also needed to clarify the role of the intracellular eyespot-bearing algae in these ciliates.

**Electronic supplementary material:**

The online version of this article (10.1186/s12862-019-1450-z) contains supplementary material, which is available to authorized users.

## Background

The study of ciliated protists, a highly differentiated and diverse group of eukaryotic microorganisms, has provided many important insights into cell biology, genetics, organismal development and evolution, biogeography, and ecology [1]. Hypotrichid ciliates (subclass Hypotrichia s. str.), as the most complex and highly differentiated members of this group, have been a focus of research in ciliatology [2–5]. However, from the systematic standpoint, it is still one of the most ambiguous group of ciliates [6]. Psilotrichidae Bütschli, 1889, a family of morphologically curious hypotrichs, is characterized by long and sparse cirri, a rigid cortex and an oral primordium developing in a deep pouch as in euplotids [7–9], not as in typical hypotrichids [10]. Members of the family have had a confused nomenclatural and taxonomic history, having been classified by various authors in different families. This is, in part, because descriptions for most of them are based only on living observations and some diagnostic features are lacking [5, 7, 11–15]. Most recently, Heber et al. [7] redefined the family Psilotrichidae and added a new genus *Psilotrichides* Heber et al., 2018, with *P. hawaiiensis* as the type species [16]. In this revision, the type species *Hemiholosticha viridis* Gelei, 1954 and *Psilotricha viridis* sensu Kahl (1932) were included in the genus *Hemiholosticha*. An accurate map of the cirral pattern of *Psilotricha viridis* sensu Kahl (1932) is not available as it was described only on the basis of live observation. A population of ciliates, described herein, was recovered from the island of Guam, and considered to be very likely conspecific with *P. viridis* sensu Kahl (1932), thus, a new species, *Hemiholosticha kahli* nov. spec., is proposed. To date, molecular data have been available for only one psilotrichid species, *Urospinula succisa* (Müller, 1786) Esteban et al., 2001. Thus the phylogeny of this group is far from being resolved.

In this work, the first records of 18S rRNA gene sequences of two psilotrichid species, *Hemiholosticha kahli* nov. spec. and *Psilotrichides hawaiiensis*, collected from Guam, are provided, and the phylogenetic analyses based on 18S rRNA gene data are presented. Detailed redescriptions of these two psilotrichid species are given based on morphological and morphometric studies.

## Results

### Taxonomy

#### *Hemiholosticha kahli* nov. spec. (Figs. 1, 2 and 5a, c; Table 1 Additional file 1: Video S1)


**Additional file 1:** **Video S1.** Live view of *Hemiholosticha kahli* nov. spec. and *Psilotrichides Hawaiiensis*. Incidentally included, *Drepanomonas* sp. and *Idiometopus turbo*. (MOV 48619 kb)


##### ZooBank registration number of present work

urn:lsid:zoobank.org:pub:D6C195E2-E71A-4D05-B3D6-534EBE0A80C1.

**Fig. 1:**
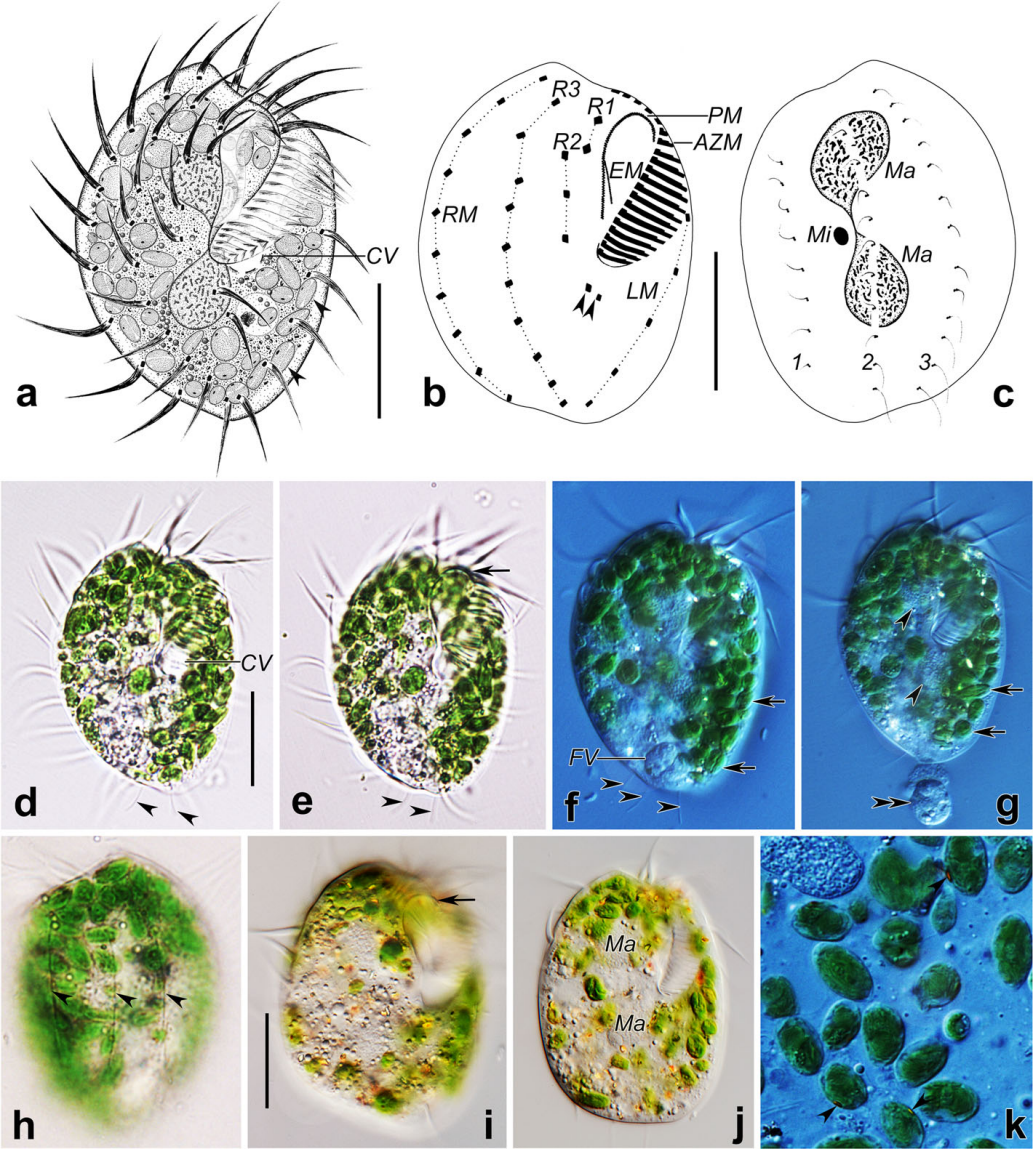
*Hemiholosticha kahli* nov. spec. in vivo (a, d–k) and after protargol impregnation (b, c). **a** Ventral view of a representative individual, arrowheads show the algae. **b, c** Ventral (b) and dorsal (c) views of a representative specimen, showing ciliature and nuclear apparatus, arrowheads show the postoral cirri, hatched lines show cirri originating from the same cirral anlage. **d–g** Ventral views of representative individuals, showing the contractile vacuole, arrowheads in (d–f) indicate the long posterior dorsal bristles, arrowheads in (g) show the macronuclear nodules, arrow in (e) shows the distinctly curved paroral membrane, arrows in (f, g) show the green algae, double arrowheads show the food discharged from the food vacuole in (f). **h** Dorsal view, showing the dorsal ribs (arrowheads). **i, j** Ventral views of a slightly squeezed specimen, showing the distinctly curved paroral membrane (arrow), macronuclear nodules and the granules. **k** Details of the green algae, arrowheads indicate the red eyespots. AZM, adoral zone of membranelles; CV, contractile vacuole; EM, endoral membrane; FV, food vacuole; LM, left marginal row; Ma, macronuclear nodules; Mi, micronucleus; PM, paroral membrane; RM, right marginal row; R1–3, ventral rows; 1–3, dorsal kineties. Scale bars: 25 μm

**Fig. 2:**
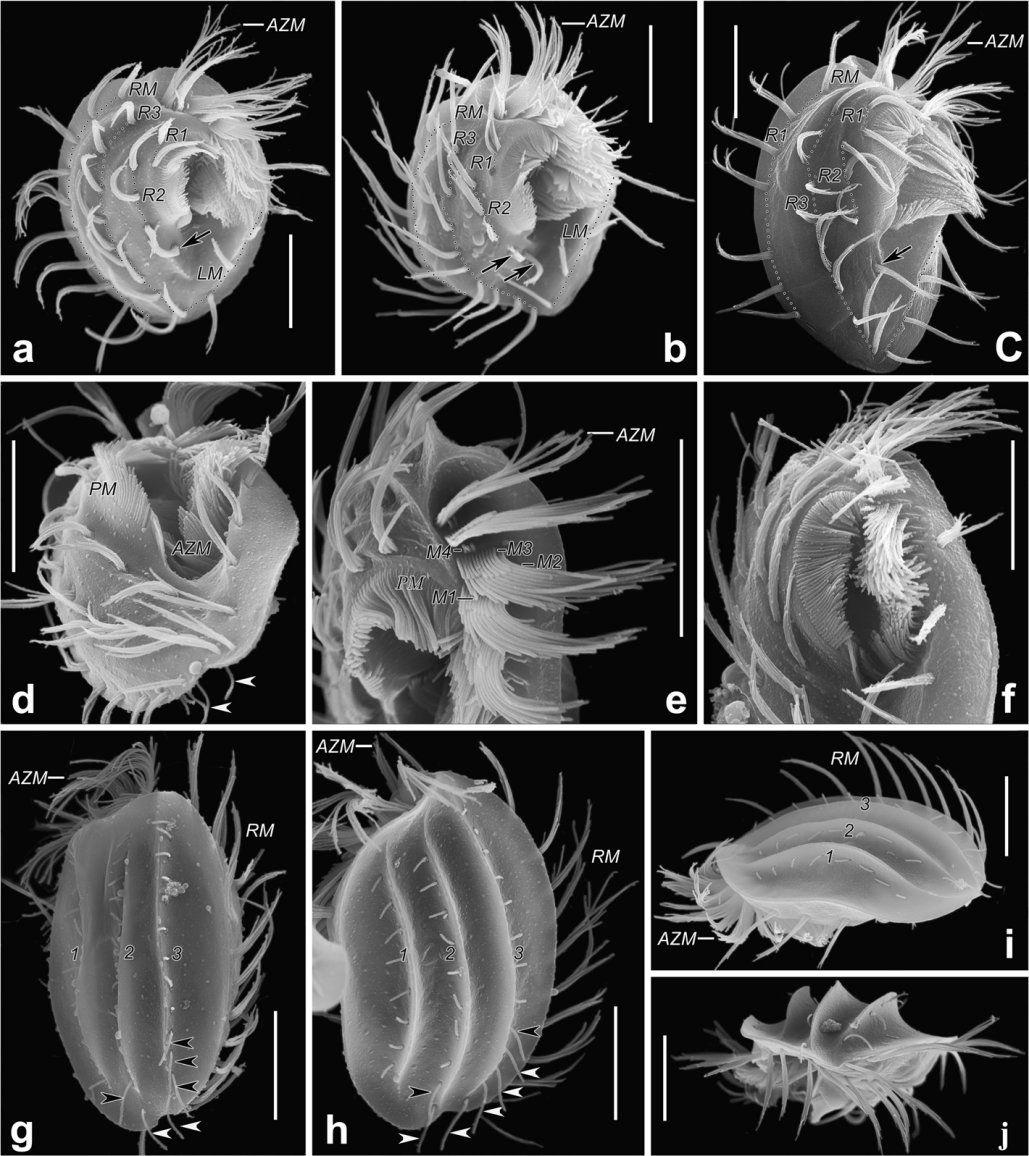
*Hemiholosticha kahli* nov. spec. in the scanning electron microscope. **a–d** Ventral views of representative individuals, showing cirral pattern, arrows indicate postoral cirri, arrowheads show the long posterior dorsal bristles, hatched lines show cirri originating from the same cirral anlage. **e, f** Details of anterior end of cells, showing structure of paroral membrane and adoral membranelles. **g, h** Dorsal views of representative individuals, showing the sharp ribs and dorsal kineties, arrowheads indicate the long posterior dorsal bristles. **i** Lateral view, showing the convex dorsal side and sharp ribs on dorsal side. **j** View from posterior body end, showing the sharp ribs on dorsal side. AZM, adoral zone of membranelles; LM, left marginal row; M1–4, ciliary rows of adoral membranelles; PM, paroral membrane; RM, right marginal row; R1–3, ventral rows; 1–3, dorsal kineties. Scale bars: 15 μm

**Table 1:** Morphometric characterization of *Hemiholosticha kahli* nov. spec. (upper line) and *Psilotrichides hawaiiensis* (lower line)

Character^a^	Mean	M	SD	CV	Min	Max	*n*
Body, length	66.5	65.0	5.3	8.0	58.0	78.0	21
	43.2	43.0	4.5	10.5	36.0	54.0	21
Body, width	48.0	47.0	3.8	7.9	42.0	56.0	21
	28.3	27.0	2.8	10.1	24.0	35.0	21
Body length: width, ratio	1.4	1.4	0.0	3.3	1.3	1.5	21
	1.5	1.5	0.1	5.5	1.4	1.7	21
Macronuclear nodules, number	2.0	2.0	0.0	0.0	2.0	2.0	21
	2.0	2.0	0.0	0.0	2.0	2.0	21
PE of anterior Ma to AE of cell, distance	10.7	11.0	1.4	13.4	8.0	14.0	21
	8.2	8.0	1.8	22.1	5.0	11.0	21
Anterior macronuclear nodule, length	17.4	17.0	1.8	10.2	14.0	21.0	21
	11.3	11.0	1.5	13.2	9.0	15.0	21
Anterior macronuclear nodule, width	11.5	11.0	1.0	8.5	10.0	13.0	21
	9.4	9.0	1.0	10.4	8.0	11.0	21
Micronuclei, number	1.0	1.0	0.0	0.0	1.0	1.0	21
	1.0	1.0	0.0	0.0	1.0	1.0	16
Micronucleus, length	3.3	3.0	0.4	12.3	3.0	4.0	21
	2.0	2.0	0.0	0.0	2.0	2.0	16
Micronucleus, width	2.5	2.5	0.3	12.6	2.0	3.0	21
	2.0	2.0	0.0	0.0	2.0	2.0	16
Adoral zone, length	33.3	34.0	2.8	8.5	30.0	41.0	21
	20.4	20.0	1.4	6.7	18.0	23.0	21
Adoral zone, percentage of body length	50.1	50.7	2.0	4.0	45.1	53.1	21
	47.6	47.6	3.6	7.6	38.9	52.6	21
Adoral membranelles, number	20.5	21.0	0.7	3.3	19.0	22.0	21
	22.0	22.0	1.0	4.7	20.0	23.0	18
Adoral membranelles, length of widest base	10.1	10.0	0.9	8.4	9.0	12.0	21
	4.2	4.0	0.2	5.8	4.0	4.5	21
Buccal cavity, width	10.7	10.0	2.6	24.7	8.0	18.0	21
	5.5	5.0	0.9	17.1	4.0	7.0	17
AE of paroral to AE of cell, distance	6.1	6.0	1.9	31.0	3.0	10.0	21
	6.3	6.0	1.1	17.5	4.0	8.0	21
Paroral, length	23.4	23.0	2.5	10.5	20.0	30.0	21
	12.3	12.0	1.5	12.4	10.0	16.0	21
AE of endoral to AE of cell, distance^b^	8.5	9.0	1.3	15.1	6.0	11.0	21
Endoral, length^b^	10.1	10.0	1.6	15.4	8.0	14.0	21
Left marginal row, number of cirri	5.1	5.0	0.6	11.1	4.0	6.0	21
	3.0	3.0	0.0	0.0	3.0	3.0	21
Postoral row, number of cirri	1.6	2.0	0.5	32.8	1.0	2.0	16
	1.7	2.0	0.5	27.7	1.0	2.0	20
Ventral row 1, number of cirri	2.0	2.0	0.0	0.0	2.0	2.0	20
	2.0	2.0	0.0	0.0	2.0	2.0	20
Ventral row 2, number of cirri	3.0	3.0	0.0	0.0	3.0	3.0	16
	1.1	1.0	0.3	28.0	1.0	2.0	20
Ventral row 3, number of cirri	9.4	9.5	0.8	8.3	8.0	11.0	18
	3.3	3.0	0.6	17.3	2.0	4.0	20
Ventral row 4, number of cirri^b^	3.4	3.0	0.5	14.6	3.0	4.0	20
Right marginal row, number of cirri	10.4	10.0	0.7	6.5	9.0	12.0	21
	4.6	4.5	0.6	13.3	4.0	6.0	20
Dorsal kinety 1, number of bristles	7.5	8.0	0.9	12.4	6.0	9.0	21
	7.5	7.0	0.8	10.1	7.0	9.0	20
Dorsal kinety 2, number of bristles	10.4	11.0	1.2	11.7	7.0	12.0	14
	7.4	7.0	0.5	6.8	7.0	8.0	17
Dorsal kinety 3, number of bristles	12.9	13.0	1.0	8.1	11.0	15.0	20
	12.7	12.0	1.4	11.2	10.0	15.0	20
Cirri, total number	31.4	31.0	1.4	4.3	29.0	33.0	15
	19.0	19.0	1.2	6.2	17.0	21.0	20

^a^All data are based on protargol-impregnated specimens. Measurements in μm

^b^Data for *Psilotrichides hawaiiensis*

Abbreviations: *AE* anterior end, *AZM* adoral zone of membranelles, *CV* coefficient of variation in %; *M* median, *Ma* macronuclear nodule, *Max* maximum, *Mean* arithmetic mean, *Min* minimum, *n* sample number, *PE* posterior end, *SD* standard deviation

##### *ZooBank registration number of Hemiholosticha kahli* nov. spec.

urn:lsid:zoobank.org:act:18DA1580-F25B-4A57-93BE-C855FCAFFF82.

##### Dedication

We dedicate this species to Alfred Kahl in recognition of his significant contributions to the taxonomy of ciliates and also the first record of the species.

##### Type locality and ecology

The sample containing *Hemiholosticha kahli* nov. spec. was collected from a stagnant freshwater roadside puddle on the east side of Rte. 4 (13°25′46.03″N, 144°46′56.97″E) on the US island territory of Guam.

##### Diagnosis

Middle-sized freshwater psilotrichid ciliate, body broadly oval in outline. Dorsoventrally flattened about 3:1, ventral side flat, dorsal side convex with 3 sharp ribs. Adoral zone occupying about half of body length, on average composed of 21 membranelles. Cirri in three ventral, one postoral, and one right and one left marginal row. Three dorsal kineties with some elongated dorsal bristles posteriorly. Two macronuclear nodules with one micronucleus in between.

##### Type specimens

One permanent slide containing the protargol-impregnated holotype specimen with registration number of LXT20160701–1 is deposited in the Laboratory of Protozoology, Ocean University of China, and one permanent protargol-impregnated paratype slide is deposited in the Natural History Museum (Registration no. NHMUK 2019.4.24.2).

##### 18S rRNA gene sequence

The length is 1663 (bp), GC content 45.58% and GenBank accession number MK211833.

##### Description based on Guam population

Cell size 50–75 × 40–55 μm in vivo (*n* = 5) and 58–78 × 42–56 μm, about 67 × 48 μm on average in protargol preparations (*n* = 21). Body rigid, oval in outline, posterior end slightly narrower, more or less conspicuous protrusions on anterior right part and posterior left part (Fig. 1a, d–g and Fig. 2a–c, g). Dorsoventrally flattened about 3:1, ventral side almost flat, dorsal side convex with 3 sharp ribs, cell margin extremely thin (Fig. 1h and Fig. 2g–j). Nuclear apparatus almost in the central quarters of cell, almost in midline of body, invariably composed of two macronuclear nodules, one micronucleus between macronuclear nodules. Macronuclear nodules ellipsoidal, close to each other, usually connected by fine strand, on average 17 × 11 μm in protargol preparations (Fig. 1a, c, g, j). Micronucleus globular to broadly ellipsoidal, on average 3.3 × 2.5 μm in protargol preparations (Fig. 1c). One contractile vacuole dorsally near body center, at the level of cytopharynx, about 8 μm in diameter in diastole (Fig. 1a, d). Cortex inflexible and colorless. Cortical granules absent. Cytoplasm colorless, contains numerous green algae (about 4–7 um in size), crystals (1–2 μm in size), and lipid droplets (1–2 μm across), algae render cells greenish (Fig. 1d–k). Intracellular green algae with peripheral red eyespot, pack almost entire cell, only absent at location of macronuclear nodules, thin cellular margin (Fig. 1a, d–k). Locomotion by moderately fast crawling on substrate or swimming while rotating around long body axis.

Total of 29–33 cirri in three ventral, one postoral, one right, one left marginal row. Consistently two cirri in ventral row 1, three in row 2; one or two postoral cirri; eight to eleven cirri in ventral row 3; four to six left marginal cirri, nine to twelve right marginal cirri (Table 1). Anterior cirrus of ventral row 1 located near distal end of adoral zone. Anteriormost cirrus of ventral row 2 positioned slightly behind posterior cirrus of ventral row 1. Cirri of ventral row 1, row 2 located above level of proximal end of adoral zone. Left marginal row begins at level of middle of adoral zone; right marginal row begins at anterior end of body. The anteriormost cirrus of ventral row 3 located slightly behind anteriormost cirrus of right marginal row. Marginal rows, ventral row 3 arranged in arcs, terminate at rear end of body. All cirri thin, long, widely spaced, with cilia about 15–20 μm long in vivo (Fig. 1a, b, d, e and Fig. 2a–c). Frontal, buccal, transverse cirri absent. Three dorsal kineties (DK), anterior ends almost reaching anterior end of cell. DK1 slightly shortened posteriorly, with dorsal bristles of same length, bristles about 3–4 μm long in vivo. DK2, DK3 almost reach posterior body end, anterior dorsal bristles about 3–4 μm long in vivo, two posteriormost dorsal bristles of DK2, four or five posteriormost bristles of DK3 about 6–8 μm long in vivo, protrude beyond posterior end of cell (Fig. 1c–f and Fig. 2g–j). Caudal cirri absent.

Adoral zone of membranelles about half (45–53%) of body length in protargol preparations, on average composed of 21 membranelles, commences anteriorly near midline of body, largest bases of membranelles 9–12 μm (on average 10 μm) wide, cilia of membranelles about 20 μm long in vivo. About five membranelles located frontally, remainder located ventrally, length of membranelle cilia increases from 5 μm to 20 μm proximally to distally. Membranelles composed of four rows of cilia with obtuse distal end: 1) length of cilia of rows 1 and 2 greatly increased from right to left; 2) cilia of row 3 distinctly shorter than those of rows 1 and 2; 3) row 4 consisting of only two or three minute cilia (Fig. 2e). Buccal cavity occupies about 22% of body width, on average about 11 μm wide in protargol preparations (Table 1). Right margin of buccal cavity and paroral membrane distinctly curved (Fig. 1a, b, e, i and Fig. 2a–f). Paroral membrane polystichomonad (≥ 3 rows of basal bodies), basal bodies completely ciliated (Fig. 5a, c), cilia up to 7 μm long, longest in central part, gradually decrease to 3 μm at both ends, paroral membrane about three quarters as long as adoral zone, about 23 μm long in protargol preparations, shorter than adoral zone proximally and distally. Endoral membrane single-rowed, terminates slightly more anteriorly than paroral, about half as long as paroral (Fig. 1b).

### *Psilotrichides hawaiiensis* Heber et al., 2018 (Figs. 3, 4 and 5b, d; Table 1; Additional file 1: Video S1)

#### Voucher material

A permanent voucher slide is deposited in the Natural History Museum, London (Registration no. NHMUK 2019.4.24.1).

**Fig. 3:**
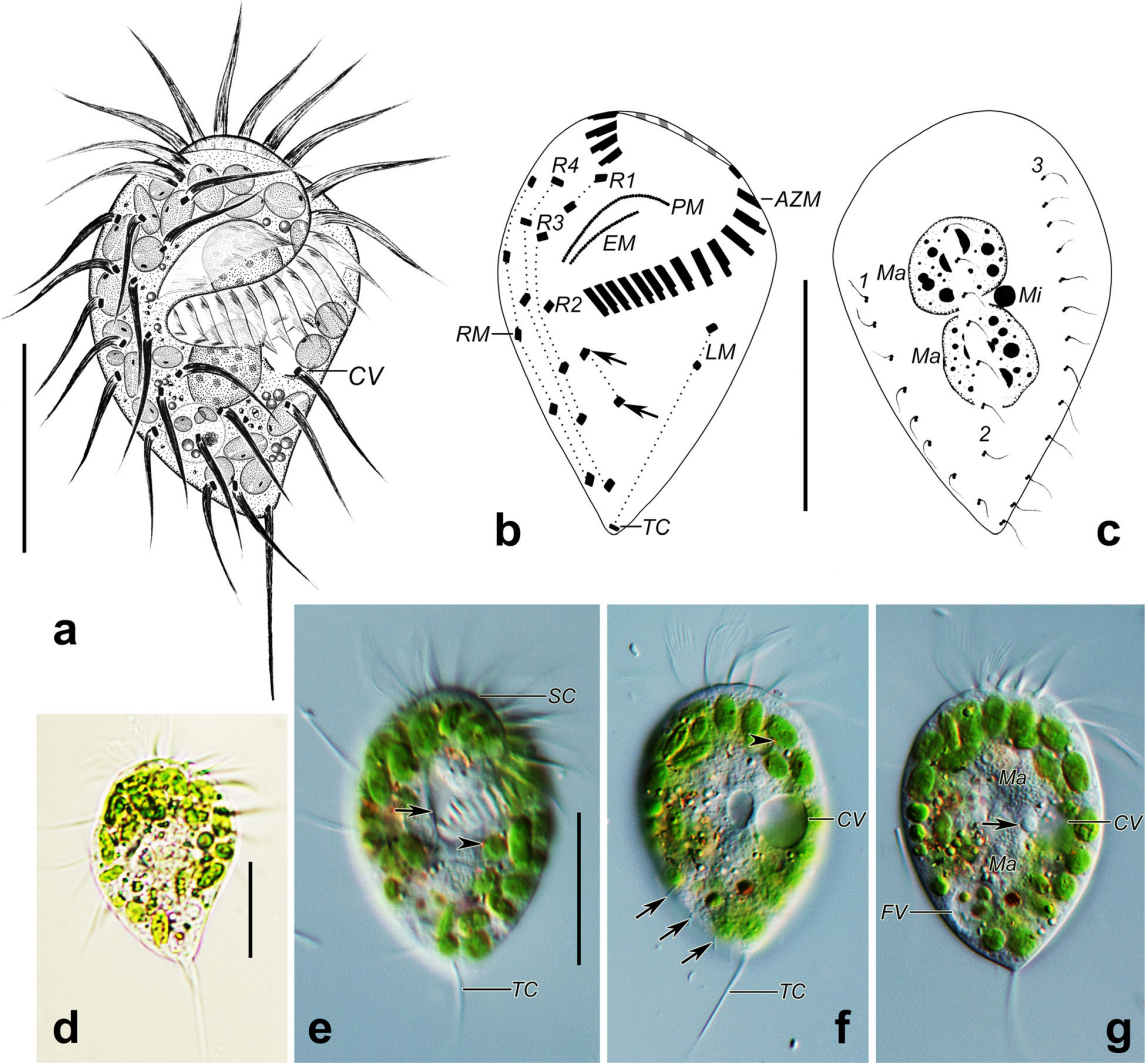
*Psilotrichides hawaiiensis* in vivo (**a**, **d**–**g**) and after protargol impregnation (**b**, **c**). **a** ventral view of a representative individual. **b, c** Ventral (**b**) and dorsal (**c**) views of a representative specimen, showing ciliature and nuclear apparatus, arrows show the postoral cirri, hatched lines show cirri originating from the same cirral anlage. **d**–**g** Ventral views of representative individuals, arrowheads show the red eyespots of the green algae, arrow in (**e**) indicates the buccal ridge, arrows in **(f**) indicate the dorsal bristles, arrow in (**g**) shows the micronucleus. AZM, adoral zone of membranelles; CV, contractile vacuole; EM, endoral membrane; FV, food vacuole; LM, left marginal row; Ma, macronuclear nodules; Mi, micronucleus; PM, paroral membrane; RM, right marginal row; R1–4, ventral rows; TC, terminal cirrus; 1–3, dorsal kineties. Scale bars: 25 μm

**Fig. 4:**
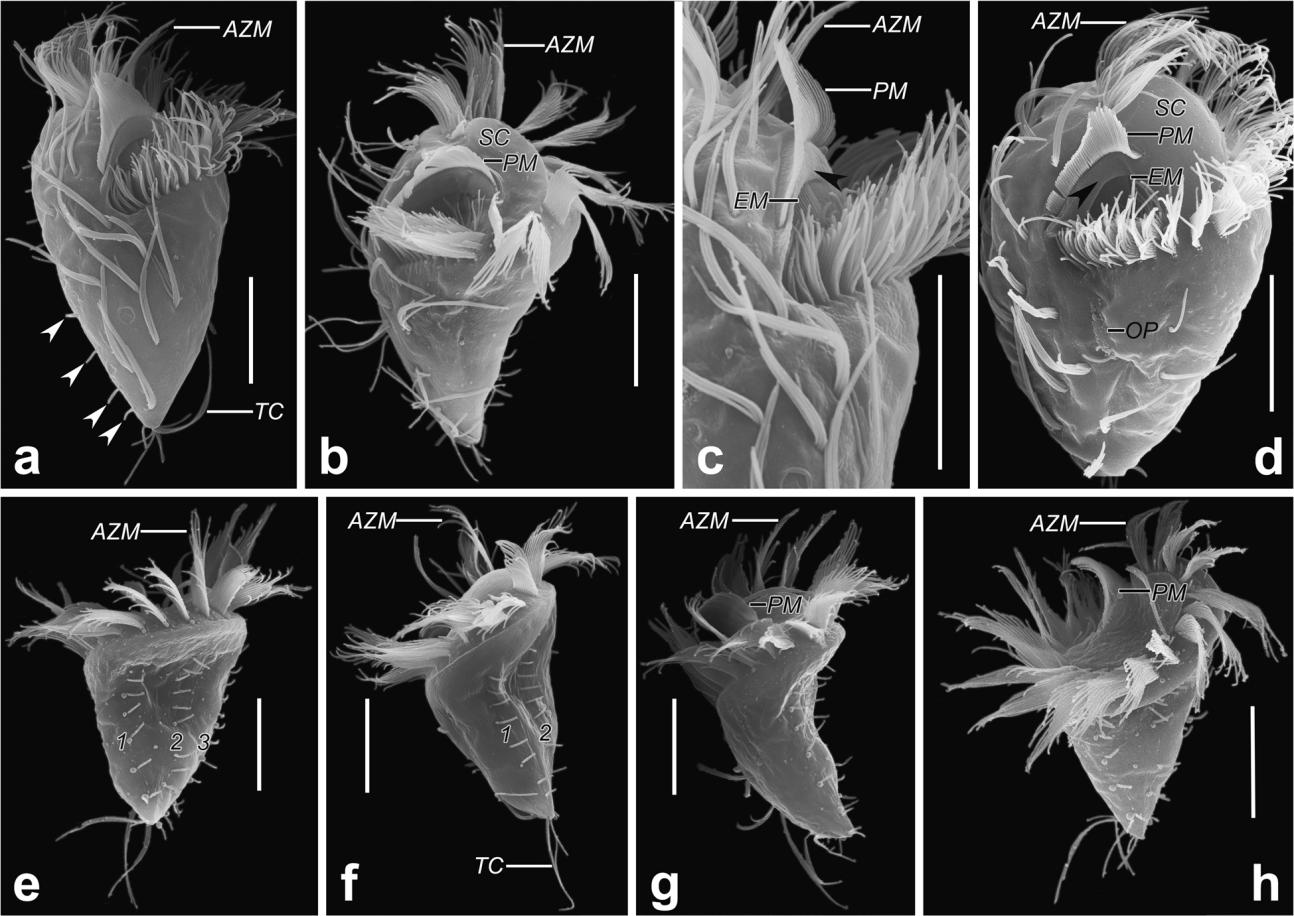
*Psilotrichides hawaiiensis* in the scanning electron microscope. **a** Ventral view of a representative individual, showing cirral pattern and the long terminal cirrus, arrowheads indicate dorsal bristles. **b** Lateral view, showing the distinctly curved paroral membrane and the obliquely truncated anterior body end. **c** Details of undulating membranes and buccal ridge (arrowhead). **d** Lateral view of a very early divider, showing the oral primordium of opisthe and structure of oral apparatus, arrowhead shows the buccal ridge. **e–h** Dorsal (e, h) and lateral (f, g) views, showing dorsal kineties, distinctly curved paroral membrane and the obliquely truncated anterior body end. AZM, adoral zone of membranelles; EM, endoral membrane; OP, oral primordium; PM, paroral membrane; RM, right marginal row; SC, scutum; TC, terminal cirrus; 1–3, dorsal kineties. Scale bars: 15 μm

**Fig. 5:**
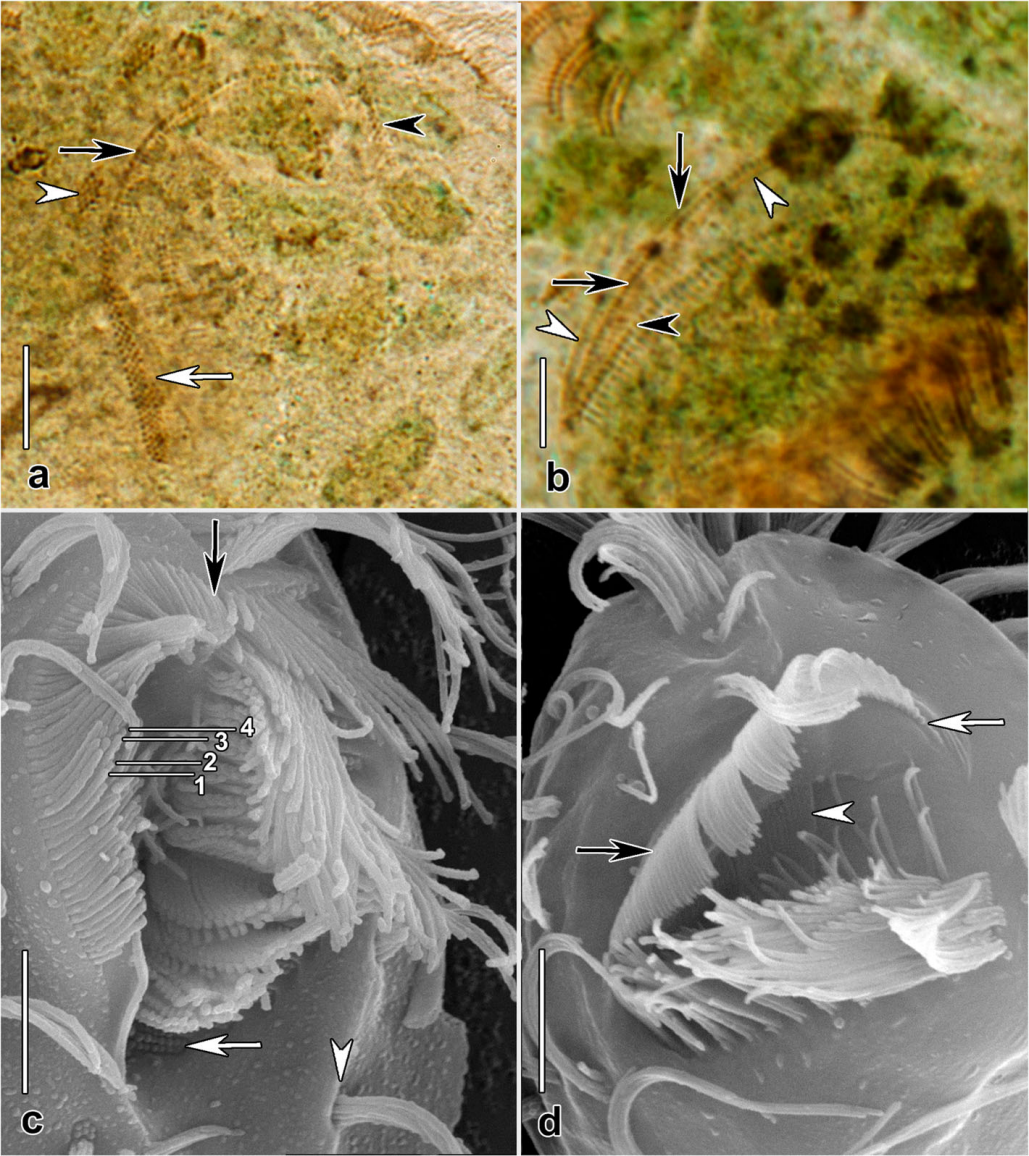
*Hemiholosticha kahli* nov. spec. (after protargol impregnation, a; in the scanning electron microscope, c) and *Psilotrichides hawaiiensis*, Guam population (after protargol impregnation, b; in the scanning electron microscope, d). **a** Ventral view showing proximal portion of polystichomonad paroral membrane with short extra row of basal bodies (white arrow), the midportion of the paroral which appears narrower due to torsion of the membrane as it follows the curve of the buccal opening (black arrow), the distal end of the paroral (black arrowhead), and the posterior cirrus of row 1 (cf. Figure 1b). **b** Ventral view showing the single file of basal bodies comprising the stichomonad paroral membrane (black arrows) and stichomonad endoral membrane (black arrowhead). The cilia of the paroral arch over the file of basal bodies from right to left causing the two parallel lines on either side (white arrowheads). **c** Ventral view showing the distal end of the paroral membrane (black arrow), tips of cilia from four rows of the polystichomonad paroral membrane (1–4), unciliated basal bodies of an adoral membranelle (white arrow), and the second left marginal cirrus (white arrowhead). **d** Ventral view showing decreasing length of cilia at the distal end of the stichomonad paroral membrane, the distal end of the membrane (white arrow), longer cilia in the midportion of the membrane (black arrow), and cilia of the stichomonad endoral membrane deep in the buccal cavity (white arrowhead). Scale bars: 5 μm

#### 18S rRNA gene sequence

The length (bp), GC content are 1662, and 46.03 respectively. GenBank accession number MK211834.

#### Description based on Guam population

Cell size 35–55 × 25–35 μm in vivo (*n* = 3), 36–54 × 24–35 μm, 43 × 28 μm on average in protargol preparations (*n* = 21). Body semirigid, obpyriform in outline, anterior end obliquely truncated, posterior end bluntly tapered (Fig. 3a, d–g and Fig. 4a, b, e–h). Dorsoventrally flattened up to 2:1, ventral side more or less convex, dorsal side sigmoidally curved (Fig. 4a, b, e–h). Two spherical to broadly ellipsoidal macronuclear nodules close to each other, almost in the central quarters of cell in midline of body, on average 11 × 9 μm in protargol preparations (Fig. 3a, c, g). Single globular micronucleus located between two macronuclear nodules, about 2 μm across (Fig. 3c, g). One contractile vacuole, positioned at left cell margin near mid-body, about 8 μm in diameter in diastole (Fig. 3a, f, g). Cortex, cytoplasm colorless. Cortical granules absent. Green algae (about 4–7 um in size) with peripheral red eyespot distributed throughout almost entire cell except at location of macronuclear nodules, render cells greenish (Fig. 3d–g). Cytoplasm contains crystals, lipid droplets, food vacuoles. Moves slowly to rapidly, swims or glides on substrate.

Total of 17–21 cirri in four ventral, one postoral, one right, one left marginal row (Fig. 3b; Table 1). Consistently two cirri in ventral row 1, anterior one located near distal end of adoral zone. Usually only one cirrus in ventral row 2, two postoral cirri. Two to four cirri in ventral row 3, three or four cirri in row 4. Distance between two anteriormost cirri of ventral row 3 and distance between two posterior cirri of ventral row 4 often increased. Invariably three left marginal cirri, anteriormost one slightly behind level of proximal end of adoral zone, posteriormost one located at rear end of cell (Fig. 3b). Four to six right marginal cirri arranged along the right margin, anteriormost cirrus located slightly behind anteriormost cirrus of ventral row 4. All cirri long, thin, cilia 15–20 μm long in vivo, except 25–30 μm long terminal left marginal cirrus composed of cilia of various lengths (Fig. 3a, d–f and Fig. 4a–c). Cirri widely spaced, makes pattern difficult to discern. Frontal, buccal, transverse cirri absent. Three dorsal kineties (DK) with anterior ends not extending to anterior end of cell. DK1, 2 shorter than DK3. All dorsal bristles same length, 3–4 μm long in vivo (Fig. 3c, f and Fig. 4a, e–h). Caudal cirri absent.

Adoral zone of membranelles occupies 39–53% (on average 48%) of body length in protargol preparations, composed of 20–23 (on average 22) membranelles, largest membranelle base about 4 μm wide. Adoral zone semicircular when viewed apically (Fig. 4h); question mark-shaped in ventral view (Fig. 3b, e). Distalmost membranelle reaches right ventral margin, membranelles partially covered by a scutum anteriorly (Fig.3e and Fig. 4b). Length of membranelle cilia gradually decreases from about 20 μm distally to 5 μm proximally. Buccal cavity occupies about 20% body width, on average about 6 μm wide in protargol preparations (Table 1). Paroral membrane single-rowed, about 12 μm (10–16 μm) long in protargol preparations, forms acute to very acute angle with longitudinal cell axis, cilia of paroral membrane longest up to 8 μm in central part, decrease to 3 μm at both ends (Fig. 4b–d and Fig. 5b, d). Paroral, endoral membranes separated by buccal ridge, endoral membrane single-rowed, extends parallel to paroral, slightly shorter than paroral anteriorly, about 10 μm (8–14 μm) long in protargol preparations (Fig. 3b and Fig. 4c, d).

### Phylogeny (Fig. 6)

The topologies of the ML and BI trees inferred from 18S rRNA gene sequences are generally congruent with variable support values. The incongruities between BI tree and the ML tree are only appeared in the deeper branches. Therefore, only the ML topology is shown, with nodal support from both methods. In the topological trees, Stichotrichida and Sporadotrichida intermingle with each other and neither order is monophyletic. In both analyses, the available psilotrichid sequences (*Hemiholosticha kahli* nov. spec., *Psilotrichides hawaiiensis*, and *Urospinula succisa*) cluster in a fully supported clade (100% ML, 1.00 BI). However, the position of Psilotrichidae among the hypotrichid ciliates is not resolved as indicated by very low support values in ML tree and inconsistent branching in BI tree. Pair-wise sequence similarities are as follows: *H*. *kahli* and *P. hawaiiensis*, 97.8%; *H*. *kahli* and *U*. *succisa*, 98.0%; *P. hawaiiensis* and *U*. *succisa*, 99.3%.

**Fig. 6:**
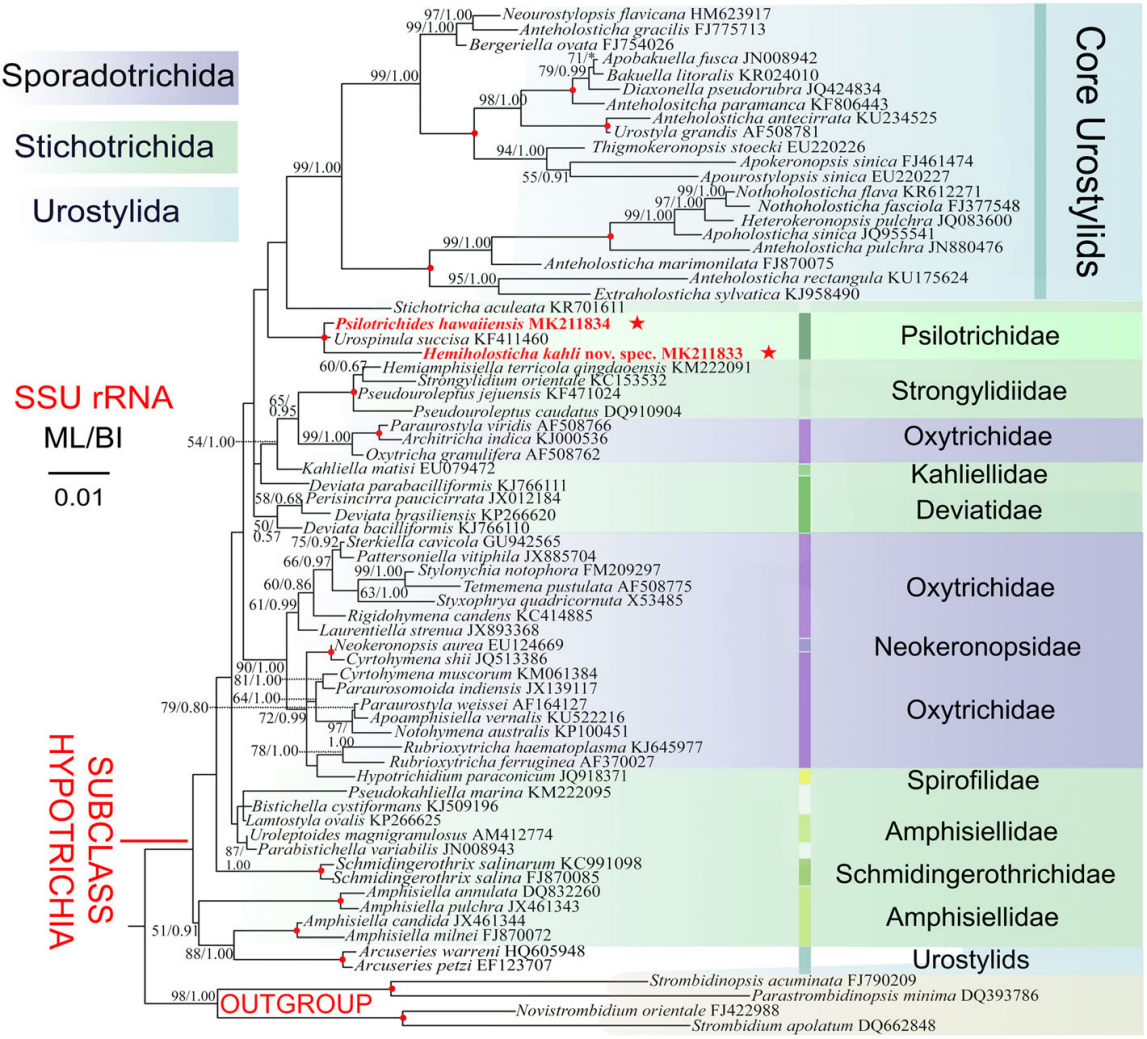
The maximum likelihood (ML) tree inferred from 18S rRNA gene sequences. Bootstrap values above 50 of maximum likelihood analysis and the posterior probability above 0.5 of Bayesian analysis are given at nodes. Fully supported (100/1.00) branches are marked with filled circles. Asterisk indicates incongruity between Bayesian inference tree and the ML tree. All branches are drawn to scale. Scale bar corresponds to 1 substitution per 100 nucleotide positions

## Discussion

### *Hemiholosticha kahli* nov. spec.

Kahl [17] described a population under the name *Psilotricha viridis* and provided only simple features observable in vivo. In 2008, Kreutz reported a Simmelried population under the name *Psilotricha viridis*, corresponding well with the population described by Kahl [17], also based only on living observations and provided detailed information on the cytoplasmic algae [18]. In the most recent revision, Heber et al. [7] assigned both *Hemiholosticha viridis* Gelei, 1954 and *Psilotricha viridis* sensu Kahl (1932) to the genus *Hemiholosticha*. Hitherto, however, no detailed information on the ciliature has been available for *Psilotricha viridis* sensu Kahl (1932), confusing the nomenclatural and taxonomic history. We agree with the classification by Heber et al. [7] and propose a new species, *Hemiholosticha kahli* nov. spec. for *Psilotricha viridis* sensu Kahl (1932), based on the study on the Guam population. The Guam population of *Hemiholosticha kahli* nov. spec. shares all of the diagnostic features with the population described by Kahl [17], including the C-shaped adoral zone of membranelles beginning near the midline of body; the distinctly curved right margin of the buccal cavity and paroral membrane; three sharp ribs on the dorsal side; and the large sized cytoplasmic algae bearing a peripheral red eyespot. Kahl [17] described two ventral rows and long dorsal bristles in the original population. He also noted that it was difficult to give the detailed cirral pattern for the species because of the algae in the cytoplasm and difficulty discerning the individual cirri. Therefore he very probably mistakenly mixed ventral row 1 and 2 (cirri of these two rows are almost arranged in a line) as a single row, and he might have regarded all the dorsal bristles as long because he observed the long posterior dorsal bristles. Compared with Kahl’s [17] illustration, the cirral pattern is almost identical with our Guam population, except for slightly more cirri in ventral row 2 and the postoral row. In consideration of the features noted in life, especially the three distinct dorsal ribs, the location of the contractile vacuole, the long dorsal bristles, and the red eyespot-bearing algae packed in the cells, the Simmelried population coincides with the population described by Kahl [17] and our Guam population. Compared to the original population described by Kahl [17], both the Simmelried population [18] and our Guam population have a relatively larger body size (83–95 μm long, 50–75 × 40–55 μm vs. 45–50 μm long). A certain variability can occur in cell size for the psilotrichid ciliates [12] and variations of body sizes can be considered as population dependent, varying with nutritional and other environmental factors. In our opinion, the minor discrepancies are not enough to separate them at species level, we regard these populations, therefore, as conspecific.

The Guam population of *Hemiholosticha kahli* nov. spec. can be distinguished from the type species *Hemiholosticha viridis* [19] by having sharp ribs on dorsal side (vs. inconspicuous ribs), a larger body size in vivo (50–75 × 40–55 μm vs. 45 × 35 μm), more adoral membranelles (19–22 vs. 13–14), more cirri (29–33 vs. 19–24), macronuclear nodules lying in a diagonal line (vs. lying along the long axis of the cell), and conspicuously long posterior dorsal bristles in DK2 and 3 (vs. short dorsal bristles).

### Identification of the Guam population of *Psilotrichides hawaiiensis*

Heber et al. [7, 16] erected a new genus and species under the name *Psilotrichides hawaiiensis*, mainly on the basis of the strongly oblique buccal cavity and undulating membranes, as well as the buccal ridge. Our obpyriform species corresponds well with the population described by Heber et al. [7] in ciliary pattern, contractile vacuole location, nuclear apparatus, the obpyriform body shape, the prominent terminal left marginal cirrus, and the unique structures of buccal apparatus, including the buccal ridge. Compared with the population described by Heber et al. [7], our Guam population has a relatively smaller body size (36–54 × 24–35 μm vs. 50–66 × 34–44 μm after protargol preparation) and less cirri (17–21 vs. 18–26). However, the ranges overlap and the minor differences can be considered population dependent, as for *Hemiholosticha kahli* nov. spec. One difference we should not ignore is that the cells of the Hawaiian population are packed with colorless, eyespot-bearing flagellates (possible *Polytoma* sp. and *Hyalogonium* sp.), while the cells of the Guam population are packed with green algae which bear a red eyespot. The difference might depend on geography and the biotopes (For more information, see next part). We therefore believe these two populations are conspecific.

### Phylogenetic analyses of the family Psilotrichidae

Members of the family Psilotrichidae Bütschli (1889) have had a confused nomenclatural and taxonomic history and have been classified in different families over the years. The systematic position of the family remains unresolved (Fig. 6). *Psilotricha*, the type genus of the family, was established by Stein [8]. Gelei [20] described the genus *Urospina*, which was changed by Corliss [21] to *Urospinula* because of preoccupation. Gelei [19] erected the genus *Hemiholosticha* in the family Oxytrichidae Ehrenberg, 1830, with *H. viridis* as the type species. Dingfelder [22] treated *H. viridis* as a junior synonym of *Psilotricha viridis* (Penard, 1922) Kahl, 1932 (original name: *Balladyna viridis* Penard, 1922). Borror [11] followed this classification and placed *Psilotricha* in the family Psilotrichidae, however, he did not include *Urospinula* in his revision. Further, Stiller [15] synonymized *Urospinula* and *Hemiholosticha* with *Psilotricha* and classified *Psilotricha* into the family Holostichidae Fauré-Fremiet, 1961. Moreover, she accepted both *Balladyna viridis* Penard 1922 and *H. viridis* Gelei, 1954 as members of the genus *Psilotricha*, which resulted in secondary homonymy. Thus, she replaced *H. viridis* Gelei, 1954 by a nomen novum: *Psilotricha geleii*, and treated *P. viridis* sensu Dingfelder, 1962 as a synonym, which was followed by Esteban et al. [12]. Esteban et al. [12] accepted both *Urospinula* and *Psilotricha* and synonymized *Hemiholosticha* with *Psilotricha*, while classifying them in different families: *Urospinula* into the family Orthoamphisiellidae Eigner, 1997 and *Psilotricha* into the family Oxytrichidae. Corliss [23] accepted the three genera, *Hemiholosticha*, *Psilotricha* and *Urospinula*, and classified the former two in the family Psilotrichidae, which was accepted by Lynn [14]. Corliss [23] classified *Urospinula* in the family Spirofilidae Gelei, 1929, while Lynn [14] transferred *Urospinula* into the family Amphisiellidae Jankowski, 1979. Foissner [13] synonymized *Urospinula* with *Psilotricha* and assigned both *Psilotricha* and *Hemiholosticha* in the family Psilotrichidae. Jankowski [24] assigned all the three genera (*Hemiholosticha*, *Psilotricha* and *Urospinula*) in Psilotrichidae. In the most recent revision for Psilotrichidae, Heber et al. [7, 16] adopted the classification by Jankowski [24] and added a new genus *Psilotrichides* Heber et al., 2018.

So far, the family Psilotrichidae appears to be a monophyletic group in the phylogenetic trees based on 18S rRNA gene sequences, that is, the two new sequences and the only psilotrichid sequence, from *Urospinula succisa*, cluster in a fully supported clade (100% ML, 1.00 BI), which supports the most recent assignment proposed by Heber et al. [7, 16] and confirms Jankowski’s [24] classification. This placement is also consistent with the combined morphologic features they share, which are not present in any other groups of the hypotrich ciliates: 1) body almost ellipsoidal in shape; 2) cortex rigid or semirigid; 3) cirri long and sparse, arranged in several rows, frontal, buccal, and transverse cirri absent; 4) caudal cirri absent; and 5) two macronuclear nodules with one intervening micronucleus [2–7].

### The eyespot-bearing green algae: symbionts, food or something else?

Symbioses between ciliates and photosynthetic algae are common and have arisen convergently multiple times in the course of evolution [25]. In one large study [26], 23% of 118 of freshwater euplanktic ciliate species harbored intracellular algae. The presence or absence of intracellular algae has been considered an important, but somewhat controversial, taxonomic character in ciliates because not all “green” taxa host algal endosymbionts [27]. The fate of algae ingested by heterotrophic ciliates is not a simple food/symbiont dichotomy [25], but rather comprises a spectrum, from a merely intermittent association of alga and ciliate (i.e. the algae are eventually digested or egested without digestion) as seen in *Disematostoma butschli,* through kleptoplastidy (selective sequestration of functional but ephemeral photosynthetic plastids) as seen in *Histiobalantium natans* and *Perispira ovum*, to fully established endosymbioses (i.e. heritability of endosymbionts, metabolic and, possibly, genetic integration of host and symbiont) as seen in the model organism *Paramecium bursaria* [28–32].

Ciliate-algal endosymbioses have some general characteristics that distinguish them from the more widespread and diverse ciliate-prokaryote symbioses and from algal symbioses in other protist groups [25, 28]. As yet, ciliate-algal endosymbioses exclusively involve photosynthetic algal partners whereas ciliate-prokaryote symbioses involve symbionts with a wide range of metabolic capabilities. In the case of freshwater ciliates, each cell of *P. bursaria* contains symbionts of only a single species and each population has symbionts of only one genotype, whereas polymicrobial consortia are common in the case of prokaryotic symbioses [33–36]. Each algal symbiont resides in its own closely apposed perialgal vacuole membrane and does not undergo cyclosis like food vacuoles. While other protists such as foraminiferans host a wide diversity of symbionts, including chlorophycean and rhodophycean algae and dinoflagellates, the endosymbionts of freshwater ciliated protists are overwhelmingly “*Chlorella*-like” algae in the class Trebouxiophyceae, and a few are from the Chlorophyceae (e.g. *Coccomyxa* sp. in *Stentor amethystinus* and a *Scenedesmus* sp. capable of infecting *P. bursaria*). Esteban et al. [28] cite two examples of “non-*Chlorella* endosymbioses” in freshwater ciliates, *Hemiholosticha kahli* nov. spec. (formerly *Psilotricha viridis* sensu Kahl, 1932) and *Loxodes rostrum.* In addition to non-*Chlorella* endosymbionts, both cases deviate substantially from the “typical” ciliate-algal symbiosis scenario. The putative symbionts, in both cases, are polymorphic, likely representing multiple taxa (possibly including *Chlamydomonas* spp. in the case of *H. kahli* and other chlorophyceans in *L. rostrum*). Characteristics of kleptoplastidy (retained chloroplasts in the ciliate cytoplasm) are absent in both cases. Kahl [17] noted eyespots in the intracellular algae of *H. kahli*. The Simmelried population of *H. kahli* contained multiple green algal morphotypes bearing orange-red eyespots and also colorless flagellates with eyespots, some within large vacuoles ([18], Abb. 7). The vacuoles surrounding algae in the German population are more typical of food vacuoles than perialgal vacuoles which are usually almost indiscernible from the algal cell wall in the light microscope. At least some of the “colorless” flagellates appear to be green forms in various stages of digestion ([18], Abb. 80, p). The Guam population of *H. kahli* contained at least two eyespot-bearing green algal morphotypes (large ellipsoidal and smaller spherical forms) but no colorless forms. Rather than hosting algal endosymbionts, *Hemiholosticha kahli* nov. spec. may be a species with high prey selectivity, like *Perispira ovum* which feeds exclusively on *Euglena proxima* [29]. The large number of intracellular algae may reflect a slower digestion time of the thick-walled algal cells. In the Guam *H. kahli*, the intracellular algae do not show such obvious evidence of digestion but do decrease in size from the anterior to posterior of the ciliate suggesting possible cyclosis and digestion of ingested algae (Fig. 1j).

In the type population of *Psilotrichides hawaiiensis*, cells contained an abundance of two colorless, eyespot-bearing flagellates (*Hyalogonium* and *Polytoma* spp.), presumably ingested as food [7]. The Guam population of *Psilotrichides hawaiiensis* harbors numerous ellipsoidal and spherical eyespot-bearing green algae, some clearly in food vacuoles (Fig. 3g). Both types of algal cells are indistinguishable from those in *H. kahli* from the same site. If the Guam *Psilotrichides* truly hosts algal endosymbionts, conspecificity with the Hawaiian population would be doubtful (it is highly unlikely that the non-photosynthetic flagellates of the Hawaiian population are symbionts). A more parsimonious explanation is that *P. hawaiiensis* also shows a food preference limited to flagellates of the Chlamydomonadales and the intracellular flagellates in both populations represent food organisms coexisting in the respective habitats. We favor the latter scenario.

A detailed discussion of *Loxodes rostrum* is beyond the scope of this article however similar considerations apply (i.e. the non-*Chlorella* intracellular algae may represent food rather than symbionts). However, it is interesting to note that the association of *L. rostrum* with green intracellular algae is rather inconsistent. Many descriptions, including the original one by Müller [37], fail to even mention them and, instead, describe the overall color of the ciliate as “gray” or “brown”, while others list the presence of intracellular algae as a diagnostic character [38–42]. In the absence of clear evidence of algal endosymbiosis in all three cases, feeding and starvation experiments, transmission electron microscopy and molecular sequencing of the intracellular algae would be helpful in elucidating their role in *Hemiholosticha kahli* nov. spec., *Psilotrichides hawaiiensis*, and *Loxodes rostrum*.

## Conclusions

In this work, we report the 18S rRNA gene sequences for species of the psilotrichid genera *Hemiholosticha* and *Psilotrichides* for the first time. The morphological classification that *Hemiholosticha*, *Psilotrichides* and *Urospinula* belong to the same family Psilotrichidae was confirmed by the molecular phylogeny as these three genera clustered in a well-supported monophyletic group. Two little-known algae-bearing species, *Hemiholosticha kahli* nov. spec. and *Psilotrichides hawaiiensis* collected from the same puddle of Guam, expand the knowledge of biodiversity and biogeography of this group of ciliates. Comprehensive discussions on the role of the intracellular eyespot-bearing algae are provided. Further morphologic and molecular studies of the intracellular algae would be helpful in elucidating their role in these ciliates.

## Methods

### Sample collection, observation and terminology

A sample containing both *Hemiholosticha kahli* nov. spec. and *Psilotrichides hawaiiensis* was collected from a stagnant freshwater roadside puddle on the east side of Rte. 4 (13°25′46.03″N, 144°46′56.97″E) on the US island territory of Guam in July, 2016 and raw cultures were maintained as described by Bourland et al. [43]. Our raw cultures of both Guam hypotrichs collapsed before further study of their intracellular algae was possible. Attempts to establish pure cultures were unsuccessful.

Swimming motion, flexibility and contractility were observed in undisturbed cells in Petri dishes under the dissecting microscope. Living cells were studied at magnifications of 100–1000× with bright field and differential interference contrast microscopy. In vivo measurements were made from photomicrographs of freely swimming cells at magnifications of 400–1000× using calibrated software (Spot imaging software, Diagnostic Instruments, Inc., USA). The protargol impregnation method of Wilbert [44] was used to reveal the infraciliature and nuclear apparatus. Counts and measurements of protargol-impregnated specimens were made directly with an ocular micrometer. Specimens for scanning electron microscopy were fixed with a 1:1 solution of 2.5% glutaraldehyde and 2% osmium tetroxide, dried in a critical point dryer EMS 850, Electron Microscopy Sciences, Hatfield, PA, USA), sputtered with gold in an Agar sputter coater (Electron Microscopy Sciences, Hatfield, PA, USA), and examined at 15 kV in a Hitachi S-3400 N scanning electron microscope (Hitachi High-Technologies Corporation, Tokyo, Japan). Drawings of protargol impregnated specimens and live cells were performed with the help of a drawing attachment and photomicrographs, respectively. Terminology is according to Heber et al. [7] and Lynn [14].

### DNA extraction, amplification and sequencing

Single cells were selected from raw samples, washed three or four times in sterile mineral water, placed individually in 0.2 ml PCR tube with 25 μl of EB buffer (Qiagen, Valencia, CA, USA) and stored at − 20 °C. Cells were not starved prior to selection. DNA was extracted from each of five cells using a modified Chelex method [45]. PCR was done as follows: in 0.2 ml PCR tubes, we used 12.5 μL GoTaq® Green Master Mix, 2× (Madison, WI, USA), 1.25 μl each of universal eukaryotic forward primer EUK-A and reverse primer EUK-B [46], both in a final concentration of 0.4 μM, and 10.5 μl of the Chelex extraction for a total volume of 25 μl. PCR was performed in the iCycler™ Thermal Cycler and DNA sequencing in both directions was done at GENEWIZ (South Plainfield, NJ, USA) as previously described [47]. Contigs were assembled by Seqman (DNAStar).

### Phylogenetic analyses

To determine the systematic position of *Hemiholosticha kahli* nov. spec. and *Psilotrichides hawaiiensis*, the 18S rRNA gene sequences of each taxon and those of 68 representative taxa from the subclass Hypotrichia, downloaded from GenBank database, were selected to construct phylogenetic trees, four oligotrichous ciliates, which are the most closely related group/sister group of hypotrichs in phylogenetic analyses [48], were chosen as outgroup taxa, (see Fig. 6 for accession numbers). All sequences were aligned in GUIDANCE with the MUSCLE alignment algorithm and ambiguous columns in the alignment were removed with default parameters (below 0.93) using the GUIDANCE2 server [49]. Both primer sequences were removed using the program BIOEDIT 7.2.5 [50]. The final alignment used for phylogenetic analyses included 1644 sites and 70 taxa. The program MrModeltest v.2.0 [51] selected the GTR + I + Γ (general time reversible + invariable sites + gamma) as the best model with Akaike Information Criterion (AIC), which was then used for both Maximum likelihood (ML) and Bayesian inference (BI) analysis. ML analysis, with 1000 bootstrap replicates, was carried out using RAxML-HPC2 on XSEDE v. 8.2.9 [52] on the CIPRES Science Gateway (URL: http://www.phylo.org/sub_sections/portal). [53]. BI analysis was performed with MrBayes 3.2.6 on XSEDE [54], with 1,000,000 generations, a sampling frequency of 100, and a burn-in of 2500 trees. The remaining trees were used to calculate the posterior probabilities using a 50% majority rule consensus. Tree topologies were visualized using SeaView v 4.6.1 [55] and MEGA 6.0 [56]. The systematic classification mainly follows Lynn [14], Heber et al. [7], Gao et al. [48], and Adl et al. [57].

## Data Availability

Sequence data are available in GenBank (Accession Numbers: MK211833, MK211834). The datasets used and/or analysed during the current study are available from the corresponding author on reasonable request. One permanent slide containing the protargol-impregnated holotype specimen of *Hemiholosticha kahli* nov. spec. circled in black ink, with registration number of LXT20160701–1 is deposited in the Laboratory of Protozoology, Ocean University of China, and one permanent protargol-impregnated paratype slide is deposited in the Natural History Museum, London (Registration no. NHMUK 2019.4.24.2). One permanent voucher slide with multiple protargol-impregnated individuals of *Psilotichides hawaiiensis* marked with black ink circles is deposited in the Laboratory of Protozoology, Ocean University of China (registration number of LXT20160702–1), and one permanent voucher slide is deposited in the collection of the Natural Museum, London, Registration no. NHMUK 2019.4.24.1.

## Abbreviations

18S rRNA: Small subunit ribosomal RNA
BI: Bayesian inference
bp: base pairs
DK: dorsal kinety
EB: Extraction buffer
GC: Guanine-cytosine
GTR + I + Γ: General time reversible + invariable sites + gamma model of nucleotide substitution
ML: Maximum likelihood
nov. spec.: novum species
PCR: Polymerase chain reaction
s. str.: sensu stricto
spp.: species (plural)
